# Visual-auditory differences in duration discrimination of intervals in the subsecond and second range

**DOI:** 10.3389/fpsyg.2015.01626

**Published:** 2015-10-26

**Authors:** Thomas H. Rammsayer, Natalie Borter, Stefan J. Troche

**Affiliations:** ^1^Institute of Psychology, University of BernBern, Switzerland; ^2^Center for Cognition, Learning, and Memory, University of BernBern, Switzerland; ^3^Department of Psychology and Psychotherapy, University of Witten/HerdeckeWitten, Germany

**Keywords:** duration discrimination, sensory modality, subsecond range, second range, distinct timing hypothesis, common timing hypothesis, timing mechanisms

## Abstract

A common finding in time psychophysics is that temporal acuity is much better for auditory than for visual stimuli. The present study aimed to examine modality-specific differences in duration discrimination within the conceptual framework of the Distinct Timing Hypothesis. This theoretical account proposes that durations in the lower milliseconds range are processed automatically while longer durations are processed by a cognitive mechanism. A sample of 46 participants performed two auditory and visual duration discrimination tasks with extremely brief (50-ms standard duration) and longer (1000-ms standard duration) intervals. Better discrimination performance for auditory compared to visual intervals could be established for extremely brief and longer intervals. However, when performance on duration discrimination of longer intervals in the 1-s range was controlled for modality-specific input from the sensory-automatic timing mechanism, the visual-auditory difference disappeared completely as indicated by virtually identical Weber fractions for both sensory modalities. These findings support the idea of a sensory-automatic mechanism underlying the observed visual-auditory differences in duration discrimination of extremely brief intervals in the millisecond range and longer intervals in the 1-s range. Our data are consistent with the notion of a gradual transition from a purely modality-specific, sensory-automatic to a more cognitive, amodal timing mechanism. Within this transition zone, both mechanisms appear to operate simultaneously but the influence of the sensory-automatic timing mechanism is expected to continuously decrease with increasing interval duration.

## Introduction

A common finding in time psychophysics is that temporal acuity is much better for auditorily than for visually presented stimuli (Penney and Tourret, 2005; van Wassenhove, 2009; Merchant et al., 2015). This also applies to perceived duration and duration discrimination as two aspects of interval timing. Perceived duration reflects the subjectively experienced duration of a given stimulus interval, while duration discrimination refers to the ability to discriminate the smallest possible difference in duration between two temporal intervals. A large number of studies demonstrated that, when a visual and an auditory stimulus are presented for the same physical time, the perceived duration of the auditory stimulus is longer than the perceived duration of the visual one (e.g., Goldstone and Lhamon, 1974; Walker and Scott, 1981; Wearden et al., 1998; Penney et al., 2000; Penney, 2003; Droit-Volet et al., 2004; Ortega et al., 2009). With regard to duration discrimination, the available data indicate better temporal discrimination of auditory compared to visually presented intervals (for concise reviews see Grondin, 2003; Rammsayer, 2014). A main objective of the present study was to contribute to a better understanding of the mechanisms involved in visual-auditory differences in temporal discrimination of extremely brief intervals in the range of 10s of milliseconds and longer intervals in the 1-s range.

There are two major conceptual frameworks to account for the timing of extremely brief and longer intervals: the *Common Timing Hypothesis* and the *Distinct Timing Hypothesis* (cf. Rammsayer and Troche, 2014). Broadly speaking, the Common Timing Hypothesis assumes a single, unitary timing mechanism irrespective of interval duration, whereas the Distinct Timing Hypothesis proposes two dissociable mechanisms for the timing of durations in the sub-second and second range, respectively.

The first psychophysical models of interval timing in the subsecond and second range, developed by Creelman (1962) and Treisman (1963), proposed a common timing mechanism based on neural counting. According to these models, a neural pacemaker generates pulses and the number of pulses associated with a physical time interval constitutes the internal time code of this interval. Thus, the higher the pulse rate, the better the temporal resolution of the timing mechanism will be, which is functionally equivalent to better performance on interval timing. More recent theoretical accounts of interval timing, the most well-known of which is Scalar Timing Theory (e.g., Gibbon and Church, 1984; Church, 2003; Allman et al., 2014), also assume such a unitary timing mechanism (Killeen and Weiss, 1987; Rammsayer and Ulrich, 2001; Grondin, 2010). Although direct experimental evidence for the notion of a single timing mechanism underlying duration discrimination in the subsecond and second range is difficult to obtain, some indirect evidence can be derived from the failure to detect a decrease in precision across two ranges of interval duration due to the breakpoint of an interval timing mechanism (Lewis and Miall, 2009). Such break points are to be expected if distinct timing mechanisms, with various levels of absolute precision, were used for measuring intervals of different durations (Rammsayer, 1996; Gibbon et al., 1997; Grondin, 2014).

Most likely, Münsterberg (1889) was the first to propose two distinct timing mechanisms underlying interval timing in the subsecond and second range, respectively. He assumed that durations less than approximately 300 ms can be perceived directly, whereas longer durations need to be formed by higher mental processes. Similarly, Michon (1985) put forward the idea that temporal processing of intervals longer than approximately 500 ms is cognitively mediated, whereas temporal processing of shorter intervals is “of a highly perceptual nature, fast, parallel and not accessible to cognitive control” (Michon, 1985, p. 40).

The Distinct Timing Hypothesis is supported by several studies (e.g., Rammsayer and Lima, 1991; Rammsayer and Ulrich, 2011) employing a dual-task paradigm with a temporal primary task (e.g., duration discrimination) and a secondary non-temporal cognitive task (e.g., word learning). In these studies, temporal discrimination of intervals ranging from 50 to 100 ms was not affected by the non-temporal secondary task, whereas discrimination of longer intervals in the 1-s range was markedly impaired by the same secondary task. These findings were consistent with Michon’s (1985) notion that temporal processing of extremely brief intervals can be regarded as sensory-automatic in nature and beyond cognitive control, while temporal processing of longer intervals demands cognitive resources. This pattern of results was corroborated by pharmacopsychological studies showing a differential effect of pharmacological agents on temporal discrimination as a function of interval duration. Drugs that interfere with working memory functioning, such as benzodiazepines, strongly impact performance on duration discrimination in the 1-s range without affecting the 10s-of-ms range (for a concise review see Rammsayer, 2008). Also findings from functional neuroimaging studies corroborate the concept of a sensory-automatic system for the timing of intervals in the range of 10s of milliseconds and a cognitively controlled, higher-order system for temporal processing of longer intervals (Lewis and Miall, 2003, 2006). Several more recent studies, also proceeding from Michon’s (1985) conception of two distinct timing mechanisms, implied that the transition from automatic-sensory to cognitively controlled timing lies closer to 250 ms than to 500 ms (e.g., Buonomano et al., 2009; Spencer et al., 2009).

Most studies on visual-auditory differences in duration discrimination, more or less implicitly, refer to the Common Timing Hypothesis to account for their findings (cf. Grondin, 2003; Rammsayer, 2014). Within this conceptual framework, better performance on auditory duration discrimination is generally ascribed to an increased number of pulses accumulated during a given time interval in the case of auditory compared to visual stimuli. This increased number of pulses yields finer temporal resolution and, thus, better timing accuracy for auditory compared to visual intervals.

Up to date and to the best of our knowledge, no experimental study appears to exist that directly addressed visual-auditory differences in duration discrimination against the theoretical background of the Distinct Timing Hypothesis. Therefore, the major goal of the present study was to explore whether there is evidence for the notion of different timing mechanisms underlying visual-auditory differences in duration discrimination of extremely brief intervals in the range of 10s of milliseconds and longer intervals in the 1-s range.

Our theoretical point of departure was provided by two recent studies applying a confirmatory factor analysis (CFA) approach. In their study on visual-auditory differences in temporal information processing, Stauffer et al. (2012) put forward the idea that modality-specific differences develop at the level of sensory-automatic processing, whereas higher-order cognitive temporal processing was assumed to be amodal and, thus, independent of sensory modality. This notion of an amodal mechanism for temporal processing of longer intervals is supported by the finding of similar tuning properties of neurons in the supplementary motor area to durations in the 450- to1000-ms range across sensory modalities (Merchant et al., 2013a,b).

In another, more recent CFA study on the internal structure of auditory interval timing in the subsecond and 1-s range, Rammsayer and Troche (2014) concluded that the assumption of two distinct mechanisms underlying the processing of extremely brief and longer intervals might be more appropriate than the assumption of a unitary timing mechanism. Most importantly, however, for the 1-s range, they proposed a shared influence of the sensory-automatic and the cognitive timing mechanism. This shared influence originates from the notion of a transition zone from primarily sensory-automatic to primarily cognitive temporal processing (Hellström and Rammsayer, 2002; Buonomano et al., 2009; Rammsayer and Ulrich, 2011). Within this transition zone, there may be a substantial degree of sensory-automatic and cognitive processing overlap as both mechanisms operate simultaneously. Thus, temporal processing of longer intervals in the 1-s range is assumed to be controlled by and functionally related to both sensory-automatic and cognitive temporal processing.

In the present study, we transferred these conclusions to visual-auditory differences in duration discrimination of extremely brief and longer intervals. By doing so, we arrived at two predictions. First, if the timing of longer durations involves not only cognitive processes but also depends, at least to some degree, on input from the sensory-automatic timing system, then visual-auditory differences in duration discrimination observed with extremely brief intervals should also become evident for longer intervals. Our second prediction, therefore, was that visual-auditory differences observed with longer intervals in the 1-s range can be explained by modality-specific differences in initial sensory-automatic processing. More precisely, if performance on duration discrimination of longer intervals, in fact, depends on sensory-automatic as well as cognitive processes, then the relative contribution of the sensory-automatic mechanism should become evident when performance scores on auditory (visual) duration discrimination with longer intervals are statistically controlled for performance on auditory (visual) duration discrimination obtained for extremely brief intervals. With such a methodological approach, the visual-auditory difference should decrease, or even disappear, in the adjusted performance scores for longer intervals, if modality-specific differences in duration discrimination indeed originate from the sensory-automatic level of temporal information processing. This line of reasoning, underlying Predictions 1 and 2, implies the following two assumptions: (1) It is possible to dissociate the contribution of the temporal processing of extremely brief intervals from that associated with cognitive processing of longer intervals and (2) the sensory-automatic mechanism is independent of the cognitive timing mechanism.

To test our predictions, participants performed auditory and visual two-alternative forced-choice duration discrimination tasks with extremely brief intervals in the subsecond range and longer intervals in the 1-s range. The durations of the standard intervals were 50 ms for the extremely brief and 1000 ms for the longer intervals. These standard durations were chosen because the hypothetical shift from one timing mechanism to the other is supposed to occur somewhere between 100 and 500 ms (Michon, 1985; Buonomano et al., 2009; Spencer et al., 2009). Furthermore, it should be noted that, when participants are required to judge the duration of time intervals, many of them use counting as a non-temporal auxiliary strategy. Because this auxiliary counting strategy becomes effective for measuring intervals longer than approximately 1200 ms (Grondin et al., 1999, 2004), the “long” standard duration was chosen not to exceed this critical value.

## Materials and Methods

### Participants

Twenty male and 26 female undergraduate students participated in the present study. Participants’ age ranged from 18 to 28 years (mean age ± standard deviation: 22.7 ± 2.5 years). All participants were naïve with regard to the purpose of the study and reported normal hearing and normal or corrected-to-normal vision. The study was approved by the ethics committee of the Faculty of Human Sciences, University of Bern, and all participants gave their written informed consent.

### Procedure

Temporal stimuli were auditory and visual intervals. Auditory stimuli were white-noise signals presented through headphones (Vivanco SR85) at an intensity of 63 dB(A) SPL. Visual stimuli were generated by a red LED (diameter: 0.48°, viewing distance: 60 cm, luminance: 48 cd/m^2^) positioned at eye level of the participant. Testing took place in a sound-attenuated room with constant ambient light.

Performance on interval timing for extremely brief and longer intervals was assessed by one block of auditory and one block of visual intervals for each time range. Each of these four blocks comprised 64 trials, and each trial consisted of a constant standard and a variable comparison interval presented with an interstimulus interval of 900 ms. The duration of the standard interval was 50 ms for the extremely brief intervals and 1000 ms for the longer ones. The duration of the comparison interval was varied according to the weighted up–down method (Kaernbach, 1991), an adaptive rule to estimate x.25 and x.75 of the psychometric function of each participant. With this psychophysical approach, x.25 and x.75 indicate the duration of the two comparison intervals at which the response “longer” was given with a probability of 0.25 and 0.75, respectively. Each experimental block consisted of two series of 32 trials converging to x.25 and x.75, respectively. For each series, the presentation order of the standard and the comparison interval was randomized and balanced. That way, standard and comparison intervals were presented first in 50% of the trials. Trials from both series were randomly interleaved within a block.

To estimate x.25 for the extremely brief intervals, the comparison interval was increased for Trials 1–6 by 3 ms if the participant had judged the standard interval to be longer and decreased by 9 ms after a “short” response. For Trials 7–32, the duration of the comparison interval was increased by 2 ms and decreased by 6 ms, respectively. The opposite step sizes were employed for x.75. The initial durations of the comparison interval were 15 ms below and above the standard interval for x.25 and x.75, respectively. For the discrimination of longer intervals, the initial values of the comparison interval were 500 ms and 1,500 ms for x.25 and x.75, respectively. To estimate x.25, the duration of the comparison interval was increased by 100 ms if the standard interval was judged longer and decreased by 300 ms after a “short” response. For Trials 7–32, the duration of the comparison interval was increased by 25 ms and decreased by 75 ms, respectively. Again, the opposite step sizes were employed for x.75.

Order of the four blocks was counterbalanced across participants. Prior to each block, practice trials were presented to familiarize participants with the task and to ensure that they understood the instructions. Participants were instructed to decide whether the first or the second interval was longer and to indicate their answers by pushing one of two designated response buttons. Each response was followed by visual correctness feedback presented on a monitor screen. As a psychophysical indicator of performance on duration discrimination, the difference limen (DL) was computed. Following Luce and Galanter (1963), DL was defined as half the interquartile range [(x.75 - x.25)/2]. With this performance measure, smaller DL values indicate better discrimination performance. More detailed information on our psychophysical approach can be found in Rammsayer (2012).

## Results

**FIGURE 1:**
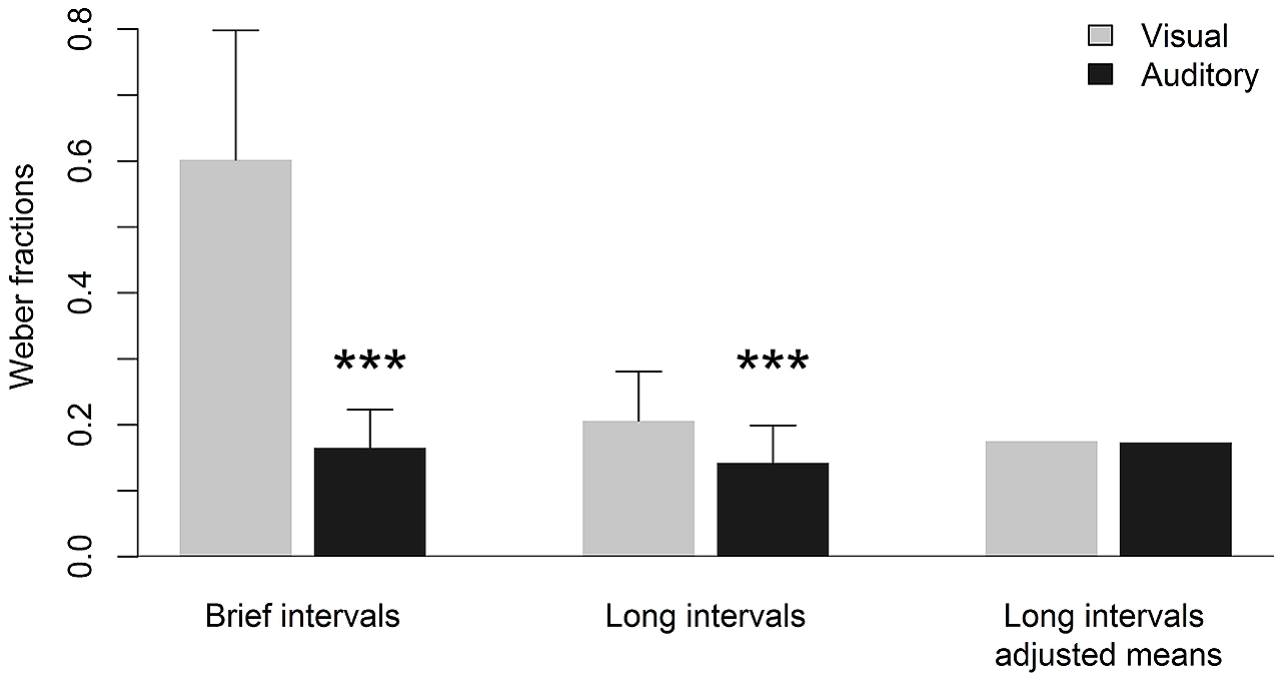
**Mean Weber fractions and standard deviations for visual and auditory duration discrimination of brief (50-ms standard duration) and longer (1000-ms standard duration) intervals as well as adjusted mean Weber fractions for duration discrimination of longer intervals.** Adjusted means represent predicted mean Weber fractions controlled for the linear effect of sensory-automatic processing in the respective sensory modality. ^∗∗∗^ significantly different from respective visual duration discrimination (*p* < 0.001).

Descriptive statistics of performance on duration discrimination as indicated by DL values are given in **Table 1**. For both extremely brief and longer intervals, smaller DL values and, thus, better performance on duration discrimination, were observed for auditory compared to visual stimuli. Subsequent *t*-tests revealed that these visual-auditory differences in DL values were statistically significant (see **Table 1**). In **Figure 1**, these visual-auditory differences are displayed graphically. For enhancing the presentation of results and to facilitate a comparison across the two ranges of interval duration, Weber fractions (DL/standard interval) are diagramed instead of absolute DL values (cf. Killeen and Weiss, 1987; Rammsayer and Grondin, 2000). The outcome of these statistical analyses is not inconsistent with our first prediction. This prediction proceeded from the assumption that temporal processing of longer intervals not only involves cognitive processes but also depends, to some degree, on input from the sensory-automatic timing mechanism. In this case, a visual-auditory difference in duration discrimination observed for extremely brief intervals in the 10s-of-ms range should also become evident for longer intervals in the 1-s range.

**Table 1:** Mean difference limen (DL) values (*M*) and standard deviations (*SD*) in ms for visual and auditory duration discrimination of brief (50-ms standard duration) and longer (1000-ms standard duration) intervals.

	Visual		Auditory		*t*	*d_z_*
				
	*M*	*SD*	*M*	*SD*		
Brief intervals	30.1	9.8	8.3	2.9	19.22^∗∗∗^	2.83
Longer intervals	206.4	74.8	141.5	56.6	5.78^∗∗∗^	0.85
						

*Also displayed are t values and Cohen’s d_z_ as effect size estimate for modality-related differences (df = 45; N = 46).^∗∗∗^ p < 0.001.*

Next, we evaluated our second prediction assuming that the visual-auditory difference in duration discrimination of longer intervals is caused by the visual-auditory difference in duration discrimination observed for extremely brief intervals. In other words, we examined whether the visual-auditory difference of longer intervals depends on the input from the sensory-automatic timing system. If this prediction is true, the visual-auditory difference of longer intervals should disappear after statistical removal of the visual-auditory effect obtained with extremely brief intervals. To test this prediction, analysis of covariance (cf. Lee, 1975; Kirk, 1995; Tabachnick and Fidell, 2013) was applied.

In general terms, this statistical approach is an extension of analysis of variance as main effects and interactions are assessed after adjusting the dependent variables for the influence of at least one covariate for each dependent variable. Thus, analysis of covariance represents a combination of regression analysis and analysis of variance. In case of a within-subject design, separate regression analyses are used to adjust each dependent variable for the influence of at least one covariate. Then, in a second step, a repeated-measurement analysis of variance is performed on the adjusted values (Tabachnick and Fidell, 2013).

Following these considerations, a repeated-measures analysis of covariance was conducted with performance on duration discrimination with auditory and visual intervals in the 1-s range as dependent variables using BMDP 2V statistical software (Dixon, 1988). Again, for enhancing the presentation of results, Weber fractions were computed and analyzed. By applying analysis of covariance, each dependent variable (in the present case: performance on visual and auditory duration discrimination of longer intervals) was adjusted for the input from the modality-specific sensory-automatic timing system as reflected by performance on visual and auditory duration discrimination of extremely brief intervals, respectively. This was achieved by two regressions. The first one regressed out visual duration discrimination of extremely brief intervals from visual duration discrimination of longer intervals, while the second one regressed out auditory duration discrimination of extremely brief intervals from auditory duration discrimination of longer intervals. The resulting adjusted means were evaluated by using the grand mean of the covariates as predictor for both regressions (for the regression equations see Kirk, 1995, p. 725). After adjusting the dependent variables for the visual-auditory difference resulting from the sensory-automatic timing system, the modality-related difference for duration discrimination of longer intervals disappeared, *F*(1,44) = 1.77, *p* = 0.19. Performance on auditory and visual duration discrimination of longer intervals was virtually identical as indicated by adjusted mean Weber fractions of 0.175 and 0.173 for auditory and visual intervals, respectively. The adjusted means of longer intervals after controlling for the influence of the sensory-automatic timing system are depicted in **Figure 1**. As the adjusted means represent the predicted means for the visual and auditory sensory modality, respectively, there is no individual variability and, thus, no standard deviations can be reported. The outcome of the analysis of covariance provided clear evidence for the notion of a modality-specific sensory-automatic timing system. When the influence of this system was statistically controlled for, a visual-auditory difference in duration discrimination of longer intervals could no longer be established.

## Discussion

The aim of the present study was to systematically investigate modality-specific differences in duration discrimination within the conceptual framework of the Distinct Timing Hypothesis. For this purpose, performance on duration discrimination of extremely brief and longer intervals in the auditory and visual modality was assessed by means of a within-subjects design. Proceeding from a modified version of the Distinct Timing Hypothesis, introduced by Rammsayer and Troche (2014), and from Stauffer et al. (2012) notion that modality-specific differences develop at the level of sensory-automatic processing rather than at the cognitive level, two predictions were made. First, if temporal processing of longer intervals in the 1-s range is, at least to some degree, dependent on input from the sensory-automatic timing mechanism, then a visual-auditory difference in duration discrimination observed for extremely brief intervals in the range of 10s of milliseconds should also become evident for longer intervals. Second, if the visual-auditory difference results from the sensory-automatic stage of temporal information processing, it should be reduced for duration discrimination of longer intervals after statistical removal of the visual-auditory effect originating from the sensory-automatic timing mechanism. Both these predictions were confirmed in the present study: superior discrimination performance for auditory compared to visual intervals could be established for extremely brief and longer intervals. However, when performance on duration discrimination of longer intervals was controlled for modality-specific input from the sensory-automatic timing mechanism, the visual-auditory difference disappeared completely as indicated by virtually identical Weber fractions for both sensory modalities.

This pattern of results is consistent with the general notion that a ‘hard’ boundary between the sensory-automatic and the cognitive mechanism is rather unlikely to exist (cf. Rammsayer and Troche, 2014). Instead, it is reasonable to assume a transition zone from one timing mechanism to the other with a significant degree of processing overlap (Hellström and Rammsayer, 2002; Buonomano et al., 2009; Rammsayer and Ulrich, 2011). With increasing interval duration, the transition from a modality-specific, sensory-automatic to a more cognitive, amodal timing mechanism gets started. Within this transition zone, both mechanisms operate simultaneously but the influence of the sensory-automatic timing mechanism is expected to decrease with increasing interval duration. This decreasing influence of the sensory-automatic timing mechanism can account for the visual-auditory difference becoming gradually smaller with increasing interval duration. Converging evidence for this notion comes from Rammsayer and Ulrich’s (2012) study where the visual-auditory difference was examined for standard durations ranging from 50 to 1400 ms. In this study, for brief standard durations below 800 ms, the visual-auditory difference, as indicated by Weber fractions, increased from 0.06 to 0.37 with standard durations decreasing from 800 to 50 ms. On the other hand, for standard durations longer than 800 ms, visual-auditory differences in Weber fractions remained almost constant at about 0.06. This gradient of visual-auditory differences in Weber fractions as a function of standard duration may be indicative of a transition from a purely modality-specific, sensory-automatic to a more cognitive, amodal timing mechanism. Moreover, these marked changes in visual-auditory differences as a function of interval duration observed in the present study and, in particular, those reported by Rammsayer and Ulrich (2012) clearly argue against the notion of a single, unitary timing mechanism as proposed by the Common Timing Hypothesis. Also neurophysiological data provided additional evidence in favor of both modality-specific and amodal mechanisms underlying the timing of intervals in the subsecond and second range (for concise reviews see Bueti, 2011; Wiener et al., 2011).

To date, the mechanism underlying the observed visual-auditory difference in duration discrimination of extremely brief intervals in the 10s-of-ms range still remains unclear. One notion refers to a finer temporal resolution due to more neural pulses accumulated with auditory intervals than with visual ones (e.g., Wearden et al., 1998; Penney et al., 2000; Droit-Volet et al., 2004). It is difficult to imagine, however, that the clock-like internal timing mechanism ticks so much faster for auditory than for visual intervals to completely account for a lowering in Weber fraction from 0.60 for visual to 0.17 for auditory intervals, as observed in the present study. This much higher temporal sensitivity in the auditory compared to the visual modality at the level of sensory-automatic temporal processing could also be due to less neural noise and, thus, faster and more accurate processing of auditory as compared to visual information (for a concise review see Stauffer et al., 2012).

A large number of studies on interval timing applying a dual-task approach support the view that processing of temporal information in the range of seconds occurs in working memory (e.g., Rammsayer and Lima, 1991; Fortin et al., 1993; Zakay, 1993; Sawyer et al., 1994; Fortin and Breton, 1995; Brown, 1997; Fortin, 1999; Rammsayer and Ulrich, 2011). Within the framework of the classical working memory model (e.g., Baddeley and Hitch, 1974; Baddeley, 1992, 2010), auditory and visual stimuli are assumed to be represented in separate and independent modality-specific stores. Quite obviously, this notion is at variance with the idea of an *amodal*, cognitive mechanism for temporal processing of longer intervals. In a most recent series of experiments, however, Salmela et al. (2014) provided experimental evidence that working memory resources are shared across representations in the auditory and visual sensory modalities. Thus, working memory can be considered a domain-general resource pool that is shared across modalities which is consistent with the basic assumption of an amodal, cognitive representation of time at a higher level of information processing (Stauffer et al., 2012; Filippopoulos et al., 2013).

Taken together, our findings are consistent with the general notion of two dissociable timing mechanisms underlying the obtained pattern of visual-auditory differences in duration discrimination of extremely brief intervals in the 10s-of-s range and longer intervals in the 1-s range: a modality-specific, sensory-automatic and an amodal, cognitive mechanism. Most importantly, however, the marked visual-auditory differences observed for duration discrimination of extremely brief intervals appeared to depend on the predominating sensory-automatic temporal processing system. Only with increasing interval duration, the amodal, cognitive timing mechanism progressively contributes to the timing process. The present study also showed that it is possible to dissociate the contribution of the sensory-automatic timing system from that of the amodal, cognitive timing system. Finally, unlike the Distinct Timing Hypothesis in its strict sense, our findings argue for a transition zone characterized by a sensory-automatic and cognitive processing overlap. From this perspective, temporal processing of longer intervals in the 1-s range seems to be controlled by and functionally related to both sensory-automatic and cognitive timing mechanisms. As the evidence that the amodal, cognitive mechanism is impacted by the modality-specific, sensory-automatic timing mechanism is based on a null result, future studies are needed to provide additional converging evidence for this notion.

## Conflict of Interest Statement

The authors declare that the research was conducted in the absence of any commercial or financial relationships that could be construed as a potential conflict of interest.
